# Comparative analysis of virulence-associated genes in ESBL-producing *Escherichia coli* isolates from bloodstream and urinary tract infections

**DOI:** 10.3389/fmicb.2025.1571121

**Published:** 2025-04-24

**Authors:** Mayuko Tanaka, Tomoko Hanawa, Tomoya Suda, Yasunori Tanji, Le Nhat Minh, Kohei Kondo, Aa Haeruman Azam, Kotaro Kiga, Shota Yonetani, Ryu Yashiro, Takuya Ohmori, Takeaki Matsuda

**Affiliations:** ^1^Department of General Medicine, Kyorin University School of Medicine, Tokyo, Japan; ^2^Department of Traumatology and Critical Care Medicine, Kyorin University School of Medicine, Tokyo, Japan; ^3^Antimicrobial Resistance Research Center, National Institute of Infectious Disease, Tokyo, Japan; ^4^Research Center for Drug and Vaccine Development, National Institute of Infectious Diseases, Tokyo, Japan; ^5^Department of Medical Technology, Faculty of Health Sciences, Kyorin University, Tokyo, Japan; ^6^Center for Data Science Education and Research, Kyorin University, Tokyo, Japan

**Keywords:** ESBL-producing *Escherichia coli*, uropathogenic *Escherichia coli*, extraintestinal pathogenic *Escherichia coli*, virulence-associated genes, urosepsis, bloodstream infections, ST131

## Abstract

The prevalence of extended-spectrum β-lactamase (ESBL)-producing *Escherichia coli* (*E. coli*) is a global health concern due to the multidrug antimicrobial resistance in extraintestinal pathogenic *E. coli* (ExPEC). ExPEC causes severe infections such as bloodstream infections, meningitis, and sepsis. Uropathogenic *E. coli* (UPEC), a subset of ExPEC, is responsible for urinary tract infections (UTIs), ranging from asymptomatic bacteriuria and cystitis to more severe conditions, such as pyelonephritis, bacteremia, and sepsis (urosepsis). Although ESBL-producing *E. coli* may have a significant impact on patient outcomes, comparisons of genotype and virulence factors between ESBL-producing and non-ESBL-producing *E. coli* have not fully elucidated the factors influencing its pathogenicity. Therefore, in the present study, we analyzed the genotypes and virulence-associated genes of ESBL-producing strains isolated from the blood of patients with UTIs to determine the characteristics of ESBL-producing UPEC strains associated with severe infections. Most of the clinical isolates belonged to phylogroup B2, with the exception of three strains from phylogroup D. The MLST was ST131, followed by ST73, ST95, and ST38, which are commonly found in UPEC strains. Intriguingly, ST131 strains were associated with fewer sepsis cases compared to non-ST131 strains (8 of 38 cases by ST131 and 5 of 8 cases by non-ST131 [OR, 0.16; 95% CI, 0.038–0.873; *p* = 0.031]). *In silico* analysis of 23 clinical isolates revealed that the genes detected in all strains may play a significant role in the pathogenesis of invasive UTIs. Clustering and gene locus analysis highlighted the genotype-MLST dependence of UPEC-specific virulence-associated genes. ST38-specific strains were atypical, characterized by the absence of several UPEC-specific genes, including *pap* loci, pathogenicity island marker (*malX*), and *ompT*, as well as the presence of genes encoding Ycb fimbriae and a Type 3 secretion system, which are typically found in enteropathogenic *E. coli* (EPEC). These results suggest that the virulence of clinical isolates causing invasive infections can vary, and that the pathogenicity of UPEC should be considered when analyzing the correlation between MLST and the repertoire of virulence-associated genes.

## 1 Introduction

Multidrug-resistant extended-spectrum β-lactamase (ESBL)-producing *E. coli* is often isolated from a variety of extraintestinal infections in humans, including bloodstream infection, lower respiratory tract infection, surgical site infection, meningitis, biliary tract infection (BTI), and urinary tract infection (UTI) (Dunn et al., 2019). In 2017, an estimated 197,400 individuals in the Unites States contracted infections caused by ESBL-producing Enterobacteriaceae, resulting in 9,100 deaths. The annual medical costs associated with these infections were projected to exceed 1 billion dollars (CDC, 2019). ESBL-producing *E. coli* is widespread in both community and healthcare settings, posing a significant public health threat due to the associated high mortality rate (Sato et al., 2024; Jernigan et al., 2020; Naghavi et al., 2024). While WHO surveillance (WHO, 2021) is ongoing, it is evident that ESBL-producing *E. coli* serves as both a relevant and representative indicator of the scope and trends in the AMR crisis. Moreover, it significantly contributes to human morbidity and mortality, while imposing a substantial economic burden on healthcare systems (Temkin et al., 2018). Furthermore, the use of carbapenem antibiotics and colistin, which are the last-line treatments for infections caused by ESBL-producing *E. coli*, has increased significantly in recent years, raising concerns about the possibility of a future lack of effective treatment options.

Uropathogenic *E. coli* (UPEC) is responsible for approximately 80% of UTIs (Foxman and Brown, 2003; Svanborg and Godaly, 1997), encompassing a diverse range of pathologies, from noninvasive infections such as asymptomatic bacteriuria and cystitis to more invasive UTIs such as pyelonephritis, which may progress to bacteremia and sepsis (urosepsis), potentially leading to life-threatening infections. UPEC produces a range of virulence factors that facilitate colonization of the urinary tract, including fimbriae, iron utilization, serum resistance, evasion of the innate immune response, and various toxins. These genetic factors significantly influence the clinical progression of UTIs. Analysis of virulence-related genes in the genomes of numerous clinical isolates (Abe et al., 2008; Wang et al., 2014; Kuhnert et al., 2000) reveals that the most representative UPEC-specific genes are primarily located within pathogenicity islands (Svanborg and Godaly, 1997; Shah et al., 2019; Terlizzi et al., 2017; Rezatofighi et al., 2021). However, the genes in the pathogenic islands vary through recombination and mobilization. Therefore, the complex pathogenesis of UPEC remains unclear (Rezatofighi et al., 2021).

The pathogenicity of ESBL-producing *E. coli* is higher than that of non-ESBL-producing *E. coli* (Lee et al., 2018; Naghavi et al., 2024). While the proportion of ESBL-producing *E. coli* isolates is increasing in both urinary tract and bloodstream infections (Ilmavirta et al., 2023), *E. coli* responsible for invasive UTIs remains poorly characterized (Pitout et al., 2005; O’Boyle et al., 2023). Although antibiograms guide antibiotic selection for UTIs, empiric therapy commonly employs penicillin or broad-spectrum cephalosporins with β-lactamase inhibitors, and fluoroquinolones (Edited by the Japanese Association for Infectious Diseases and the Japanese Society for Chemotherapy, 2023; Nelson et al., 2024). However, susceptibilities to these antibiotics are declining (WHO, 2022). In severe cases, cephamycins (e.g., cefmetazole), fosfomycin, or faropenem are considered, with carbapenems as the preferred first-line treatment (Edited by the Japanese Association for Infectious Diseases and the Japanese Society for Chemotherapy, 2023; Nelson et al., 2024).

For developing effective diagnostic and therapeutic strategies for infections caused by *E. coli* strains producing ESBLs, which are increasingly becoming difficult to treat owing to development of drug resistance, it is crucial to identify the factors associated with disease severity. Therefore, in this study, we aimed to evaluate previously reported representative UPEC specific virulence-associated genes (Johnson and Stell, 2000; Yamamoto et al., 1995) in ESBL-producing *E. coli* strains from bloodstream infections caused by UTIs, using PCR. In addition, we aimed to identify virulence-associated genes through genome sequencing and *in silico* analysis of non-ST131 and randomly selected ST131 strains.

## 2 Materials and methods

### 2.1 Cases of ESBL-producing *Escherichia coli* infections and clinical isolates

Between May 2017 and November 2022, ESBL-producing *E. coli* were prospectively collected from the blood samples as part of routine medical practices of patients visiting the Department of Emergency Medicine and Emergency General Medicine, Kyorin University Hospital. A total of 60 ESBL-producing *E. coli* strains were obtained and analyzed using bacterial colonies obtained from the first blood agar inoculum (stored at −80°C). Identification of ESBL-producing *E. coli* and drug susceptibility testing were performed using a BD Phoenix™ system (BD Diagnostics, Franklin Lakes, NJ, United States) with an NMIC/ID-441 panel and the system automatically performs assays, determines the results of MICs (Murata et al., 2023). To assess the ESBL producing-phenotype, the double-disk synergy test (DDST) was performed (CLSI [M100], 31st ed.). The first isolate from each patient was considered the primary strain, and duplicates were excluded. The primary source of infection and genotypes of the bacterial strains are shown in Table 1. After colony formation on blood agar plates from the initial blood inoculation, clinical isolates were stored at −80°C until further analysis.

**Table 1:** Primary source of infection and genotype of bacterial strains.

Primary source of infection	Total (*n* = 60) (%)	B2 (*n* = 53)	D (*n* = 6)	F (*n* = 1)
Urinary tract infection	46 (76.7)	43	3	0
Biliary tract infection	9 (15.0)	7	1	1
Others	5 (8.3)	3	2	0

In cases where bacteria detected in urine, bile, or ascites cultures matched those identified in blood cultures, the respective organ was identified as the source of infection. In cases where only blood cultures were performed, the organ with apparent infection was considered the primary site. Cases in which the infected organ could not be identified were classified as unexplained were included in others together with gastrointestinal perforation. Data are expressed as number or percent (%) of the strains.

Bacteremia was defined as the detection of bacteria in blood cultures using the BACT/ALERT ®3D (bioMérieux, Marcy-l’Étoile, France). Urine culture results were used solely as the basis for diagnosing UTIs, in conjunction with clinical symptoms. Bacterial detection in urine samples was conducted through routine examination by streaking of 2 μL of urine samples on sheep blood agar plates, followed by incubation at 37°C for 18 h. If the result was positive, colonies that formed on the sheep blood agar were further analyzed for *E. coli* identification and the ESBL-producing phenotype. Sepsis was determined based on clinical criteria: a suspected or proven focus of infection along with an acute elevation of Sequential Organ Failure Assessment score ≥2 points, which serves as a proxy for organ dysfunction, in accordance with the Japanese Clinical Practice Guidelines for Management of Sepsis and Septic Shock (2016 and 2020).

### 2.2 Genotyping by PCR and analysis of virulence-associated gene retention

*E. coli* strains were cultured in Lysogeny Broth (LB) or on LB agar at 37°C. DNA was extracted following the instructions provided with the Wizard® Genomic DNA Purification Kit (Promega, Madison, United States), according to the manufacturer’s instructions. Phylogeny determination was performed according to Clermont’s scheme (Clermont et al., 2013; Clermont et al., 2019). Representative virulence-associated genes (*papAH*, *papGII*, *papGIII*, *cnf1*, *hlyA*, *kpsMTII*, *fyuA*, *iutA*, *usp. malX*, *traT*, *ompT*, *fimH*, and *csgA*), commonly associated with UPEC, were detected by PCR. PCR was conducted using the primers listed in Table 2. The ST131 genotype was determined using a CicaGeneus® *E. coli* POT kit (Kanto Chemical Co., Ltd.), according to the manufacturer’s instructions, and negative results were considered non-ST131.

**Table 2:** PCR primers used in this study.

Target gene	Primer name	Primer sequence (5′-3′)	References
*fimH*	fimH-F	TCGAGAACGGATAAGCCGTGG	Adams-Sapper et al. (2013)
	fimH-R	GCAGTCACCTGCCCTCCGGTA	
*csgA*	csgA-F	ACTCTGACTTGACTATTACC	Darvishi (2016)
	csgA-R	AGATGCAGTCTGGTCAAC	
*papAH*	papAH-F	TGTTCAGTAATGAAAAAGAGGTTGT	This study
	papAH-R	TGAGCCGGAGGCTGAATTTT	
*papGII*	papGII AlleleII-f	GGGATGAGCGGGCCTTTGAT	Adams-Sapper et al. (2013)
	papGII AlleleII-r	CGGGCCCCCAAGTAACTCG	
*papGIII*	papGIII-F	ACGCTGAATGCCACGTAAGA	This study
	papGIII-R	TTTTGCATGGCTGGTTGTTC	
*fyuA*	FyuA f	TGATTAACCCCGCGACGGGAA	Johnson and Stell (2000)
	FyuA r	CGCAGTAGGCACGATGTTGTA	
*iutA*	AerJ f	GGCTGGACATCATGGGAACTGG	Johnson and Stell (2000)
	AerJ r	CGTCGGGAACGGGTAGAATCG	
*kpsMTII*	kpsII-F	GCGCATTTGCTGATACTGTTG	Johnson and Stell (2000)
	kpsII-R	CAATGATCGTATCGATGGGTTTT	This study
*traT*	TraT f	GGTGTGGTGCGATGAGCACAG	Johnson and Stell (2000)
	TraT r	CACGGTTCAGCCATCCCTGAG	
*ompT*	ompT_mf	TTTGATGCCCCAGATATCTATCGG	Desloges et al. (2019)
	ompT_mr	GGCTTTCCTGATATCCGGCCATG	
*cnf1*	cnf1	AAGATGGAGTTTCCTATGCAGGAG	Yamamoto et al. (1995)
	cnf2	CATTCAGAGTCCTGCCCTCATTATT	
*hlyA*	hly1	AACAAGGATAAGCACTGTTCTGGCT	Yamamoto et al. (1995)
	hly2	ACCATATAAGCGGTCATTCCCGTCA	
*usp*	usp-F	ATGCTACTGTTCCCGAGTAGTGTGT	This study
	usp-R(N7)	CATCATGTAGTCGGGGCGTAACAAT	Yamamoto et al. (1995)
*malX*	RPAi f	GGACATCCTGTTACAGCGCGCA	Johnson and Stell (2000)
	RPAi r	TCGCCACCAATCACAGCCGAAC	

### 2.3 Whole-genome sequencing and genotyping: *de novo* assembly and annotation

Clinical isolates were cultured in LB broth at 37°C for 15 h, and genomic DNA was extracted using the Wizard® HMW DNA Extraction Kit (Promega, Madison, United States), according to the manufacturer’s instructions. Libraries were prepared using the QIAseq FX DNA Library Kit (Qiagen, Hilden, Germany) according to the manufacturer’s instructions. Paired-end sequencing was performed on the DNBSEQ platform. Sequence reads were assembled *de novo* into contigs using Shovill version 1.1.0.^1^ Genomic annotation was performed using Prokka version 1.14.6.^2^ Genomic data for the 23 strains were deposited in GenBank (BioProject ID: PRJDB18240).

### 2.4 *In silico* analysis

Phylogroup, MLST, serotype, as well as FimH, and FumC type were analyzed using ClermonTyping,^3^ MLST version 2.23.0,^4^ SerotypeFinder 2.0, and CHTyper 1.0 (Center for Genomic Epidemiology^5^), respectively.

ABRicate version 1.0.1^6^ was used to detect virulence-associated genes, antibiotic-resistance genes, and plasmids using the default parameters. The databased used included *E. coli*_VF,^7^ Resfinder (Zankari et al., 2012; Feldgarden et al., 2019), and the National Center for Biotechnology Information (NCBI) AMRFinderPlus (Feldgarden et al., 2019). Protein or DNA sequence searches were performed using the Basic Local Alignment Search Tool (BLAST) at NCBI^8^ to predict gene function.

### 2.5 Clustering analysis

Cluster analysis, heatmap generation, and dendrograms construction were performed using Seaborn version 0.12.02 (statistical data visualization^9^). The reference strain for ESBL-producing ST131 UPEC included EC598 (GenBank accession No. HG941718), CFT073 (GenBank accession No. AE014075.1), UTI89 (GenBank accession No. CP000243), and 536 (GenBank accession No. CP000247). Additionally, genomes of UPEC strains from phylogroups D-38 and D-69, with assembly status from EngteroBase, were also included in the analysis (Supplementary Table S1).

### 2.6 Statistical analyses

Data were presented as counts and percentages, means with standard deviations (SD), or medians with interquartile ranges (25th–75th percentiles). Numerical and categorical variables were analyzed using the Student’s *t*-test or Fisher’s exact test. Statistical significance was set at *p* < 0.05. Odds ratio (OR) with 95% confidence intervals (CI) were also calculated. All statistical analyses were performed using the GraphPad Prism 8 software (GraphPad Software Inc., La Jolla, CA, United States).

## 3 Results

### 3.1 Genotypes, antibiotic resistance, and the set of virulence-associated genes of clinical isolates

Among the 60 bloodstream infections caused by ESBL-producing *E. coli*, 76.7% were UTIs (46 cases), with the remainder attributed to BTIs and gastrointestinal perforations (Table 1). Of the 46 UTI cases, three urine cultures were not examined due to patient conditions, and two were negative, likely due to prior antibiotic use. This study analyzed only clinical isolates from blood samples. Phylogenetic groups are summarized in Table 1. Of the UPEC strains, 43 (93.5%) belonged to phylogroup B2, while the remaining three strains were classified under phylogroup D (Table 1). Urosepsis was observed in 8 of 38 cases infected with ST131 strains and in 5 of 8 cases infected with non-ST131 strains (Table 3). The incidence was significantly lower in patients with ST131 infections compared to those with non-ST131 infections [OR, 0.16; 95% CI, 0.038–0.873; *p* = 0.031]. All strains were susceptible to imipenem/cilastatin, meropenem, cefmetazole, and latamoxef. On the other hand, approximately 80% of the strains exhibited non-susceptibility (R or I) to fluoroquinolones (Table 4). Resistance rates for ciprofloxacin and levofloxacin were 89.6 and 87.5%, respectively, for ST131 strains, compared to 75 and 50.0%, respectively, for non-ST131 strains.

**Table 3:** Characters of patients and genotypes of bacterial strains isolated from UTIs.

Baseline characteristics of the patients		UTIs total	Phylogroup B2		D
			ST131	Non-ST131	
		(*n* = 46)	(*n* = 38)	(*n* = 5)	(*n* = 3)
Male/female		15/31	14/24	1/4	0/3
Age (year)	Median (IQR 25-75)	84 (72–88)	86 (78–89)	79 (68–84)	59 (57–65)
	Min-Max	26–96	26–96	50–95	55–71
Underlying diseases	Cancer	7	6	1	0
	Diabetes mellitus	9	6	2	1
	Immunodeficiency^a^	7	6	1	0
Sepsis^b^		13	8	3	2

a Patients with chronic kidney disease (including patients undergoing dialysis), malnutrition, hypothyroidism, autoimmune diseases, and those receiving immunosuppressive or immunomodulatory agents were classified under immunodeficiency.

b In accordance with the Third International Consensus Definitions for Sepsis and Septic Shock (Sepsis-3), cases with a Sequential Organ Failure Assessment (SOFA) score of 2 or higher were diagnosed as sepsis.

**Table 4:** Antibiotic susceptibility of clinical isolates.

Antibiotics	Total (*n* = 46)		ST131 (*n* = 38)		Non-ST131 (*n* = 8)	
Ampicillin	46	(100.0%)	38	(100.0%)	8	(100.0%)
Piperacillin	46	(100.0%)	38	(100.0%)	8	(100.0%)
Ceftazidime	46	(100.0%)	38	(100.0%)	8	(100.0%)
Cefazolin	46	(100.0%)	38	(100.0%)	8	(100.0%)
Cefepime	46	(100.0%)	38	(100.0%)	8	(100.0%)
Cefmetazole	0	(0.0%)	0	(0.0%)	0	(0.0%)
Cefotaxime	46	(100.0%)	38	(100.0%)	8	(100.0%)
Cefpodoxime proxetil	46	(100.0%)	38	(100.0%)	8	(100.0%)
Cefuroxime	46	(100.0%)	38	(100.0%)	8	(100.0%)
Latamoxef	0	(0.0%)	0	(0.0%)	0	(0.0%)
Aztreonam	46	(100.0%)	38	(100.0%)	8	(100.0%)
Imipenem/cilastatin	0	(0.0%)	0	(0.0%)	0	(0.0%)
Meropenem	0	(0.0%)	0	(0.0%)	0	(0.0%)
Sulbactam/ampicillin	35	(76.1%)	29	(75.0%)	6	(75.0%)
Tazobactam/piperacillin	1	(2.2%)	1	(4.2%)	0	(0.0%)
Amikacin	0	(0.0%)	0	(0.0%)	0	(0.0%)
Gentamicin	14	(30.4%)	11	(25.0%)	3	(37.5%)
Ciprofloxacin	39	(84.8%)	33	(89.6%)	6	(75.0%)
Levofloxacin	36	(78.3%)	32	(87.5%)	4	(50.0%)
Sulfamethoxazole-trimethoprim	17	(37.0%)	13	(33.3%)	4	(50.0%)

Data are expressed as number and percent (%) of non-susceptible strains to each antibiotic.

### 3.2 Virulence-associated genes in clinical isolates analyzed by PCR

Several factors related to colonization, iron acquisition, and serum resistance contribute to UPEC pathogenicity (Terlizzi et al., 2017). We selected key UPEC virulence-associated genes for assessment via PCR (Table 5; Supplementary Table S2). The *csgA*, *fimH*, and *fyuA* genes were detected in all strains. In contrast, *malX*, *usp.* and *ompT* genes were detected in all phylogroup B2 strains but not in phylogroup D strains. Additionally, *traT* and *iutA* genes were detected in more than half of the B2 strains, while none of the phylogroup D strains tested positive for these genes. Overall, phylogroup D strains exhibited significantly fewer virulence-associated genes than phylogroup B2 strains (Table 5; Supplementary Table S2). Notably, *papAH* and *papGII* or *papGIII* genes, which are associated with P fimbriae (P pili) and the development of pyelonephritis (Ambite et al., 2019), were absent in all phylogroup D strains.

**Table 5:** Number of virulent associated genes in clinical isolates analyzed by PCR.

Virulent associated genes		Phylogroup B2		Phylogroup D (*n* = 3)	Total (*n* = 46)
		ST131 (*n* = 38)	Non-ST131 (*n* = 5)		
Type-1 pili	*fimH*	38	5	3	46
Curli fibers	*csgA*	38	5	3	46
P fimbriae	*papAH*	10	4	0	14
	*papGII*	9	3	0	14
	*papGIII*	1	1	0	
Iron utilization	*fyuA*	38	5	3	46
	*iutA*	38	4	0	42
Complement inhibition･immune evasion	*kpsMTII*	35	5	3	43
	*traT*	28	1	0	29
	*ompT*	38	5	0	43
Pathogenicity island marker	*malX*	38	5	0	43
Toxin	*cnf1*	9	2	0	11
	*hlyA*	9	1	0	10
	*usp*	38	5	0	43

The occurrence of virulence-associated genes among ESBL-producing UPEC isolates from bloodstream infections. The sum of *papGII* and *papGIII* positive strains were indicated in the total column.

### 3.3 Whole-genome analysis and characterization of clinical isolates

Fifteen ST131 strains, randomly selected from 38 identified using the POT kit, were subjected to whole-genome analysis alongside eight non-ST131 strains. This *in silico* analysis focused on genotype, serotype, FimH and FumC types, resistance genes, and the presence of pathogenic genes (Table 6).

**Table 6:** Characters of the clinical isolates obtained by *in silico* analysis and each patient data.

Strain	Phylo group	MLST	O antigen	H antigen	*fimH* type	*fumC* type	CTX-M	Age	M/F^a^	Disease
KYE006	D	38	O45	H15	24	26	14	55	F	Pyelonephritis, septic shock, DIC
KYE008	B2	131	O25	H4	30	40	27	83	F	UTI, septic shock
KYE011	D	38	O51	H40	5	26	14	71	F	UTI, septic shock
KYE013	B2	131	O25	H4	30	40	27	86	M	Pyelonephritis
KYE016	B2	1,193	O75	H5	64	14	27	68	M	UTI, septic shock
KYE019	B2	131	O25	H4	30	40	27	64	F	Renal cyst infection
KYE020	D	38	O50/O2	H30	5	26	14	59	F	Pyelonephritis
KYE024	B2	131	O25	H4	30	40	27	65	F	Renal abscess, sepsis
KYE026	B2	131	O25	H4	30	40	15	89	F	UTI
KYE027	B2	131	O25	H4	30	40	15	94	M	UTI
KYE034	B2	95	O1	H1	16	38	14	95	F	UTI
KYE035	B2	131	O16	H5	43	40	27	80	M	UTI, septic shock
KYE040	B2	131	O25	H4	30	40	15	90	F	UTI
KYE042	B2	73	O25	H1	12	24	8	84	F	UTI, septic shock
KYE047	B2	131	O25	H4	30	40	15	76	F	Pyelonephritis
KYE049	B2	131	O25	H4	30	40	15	82	F	Pyelonephritis
KYE054	B2	131	O25	H4	30	40	15	96	F	Pyelonephritis
KYE056	B2	131	O16	H5	41	40	27	88	M	Acute prostatitis
KYE057	B2	*^b^	O25	H4	30	40	27	87	M	UTI
KYE059	B2	1,193	no-hit	H5	64	14	27	79	F	UTI, sepsis
KYE060	B2	131	O25	H4	30	40	15	28	F	Pyelonephritis
KYE062	B2	131	O25	H4	54	40	27	89	M	UTI
KYE064	B2	1,193	O75	H5	64	14	27	50	F	Pyelonephritis

a Acronyms F and M stand for Female and Male, respectively.

b Single nucleotide substitution (16G → A) was detected in the adk allelic profile of ST131.

Genome analysis confirmed that the MLST results were consistent with the PCR findings, with the exception of KYE057. Although identified as ST131 using a POT kit, KYE057 was determined to belong to the ST131 lineage based on a single nucleotide substitution (16G → A) in the *adk* gene, and was included in the ST131 group for statistical analysis. Non-ST131 strains were classified as ST95, ST73, or ST1193 in phylogroup B2 and ST38 within phylogroup D. Consistent with previous studies (Pitout et al., 2022), the ST1193 strain exhibited a disruption of *lacY* due to a frameshift mutation (Supplementary Table S3). Regarding O antigen type, O25 was the most prevalent (14 strains), followed by O16 and O75, all commonly associated with UPEC. The ESBL genotypes were CTX-M27 (11 strains), CTX-M15 (7 strains), CTX-M14 (4 strains), and CTX-M8 (1 strain). Notably, the ST131-O25:H4 H30 pandemic clones were associated with CTX-M15 and CTC-M27, while the three ST38 strains were associated with CTX-M14 (Table 6).

### 3.4 Virulence-associated genes in clinical isolates analyzed *in silico*

*In silico* analysis of virulence genes using ABRicate (Supplementary Table S4) revealed that ST131 clinical isolates, including the UPEC ST131 type strain EC958, carried a significantly fewer number of virulence-associated genes compared to non-ST131 strains (<0.0001; Table 7). Well-known UPEC-specific genes, including *ompT*, *usp.* and *malX*, were detected in all phylogroup B2 strains in both PCR and *in silico* analyses. However, these genes were absent in the ST38 strains.

**Table 7:** Number of virulent associated genes analyzed *in silico* (*n* = 23).

Phylogroup	MLST	Median (IQR 25-75)	Min-Max
B2	ST131 (*n* = 15)	192 (190-200)	174-207
	Non-ST131 (*n* = 5)	220 (219-228)	209-233
D	Non-ST131 (*n* = 3)	227 (223-234)	218-240

Data are expressed as median (IQR 25–75), minimum (Min) and maximum (Max) number of virulence-associated genes detected by ABRicate were indicated. The virulence-associated genes in 536, CFT073, CFT073, UTI89, and EC958 were 261, 234, 257, and 198, respectively. The number of virulence-associated genes was tailed from the numbers of genes indicated in Supplementary Table S4.

**Figure 1:**
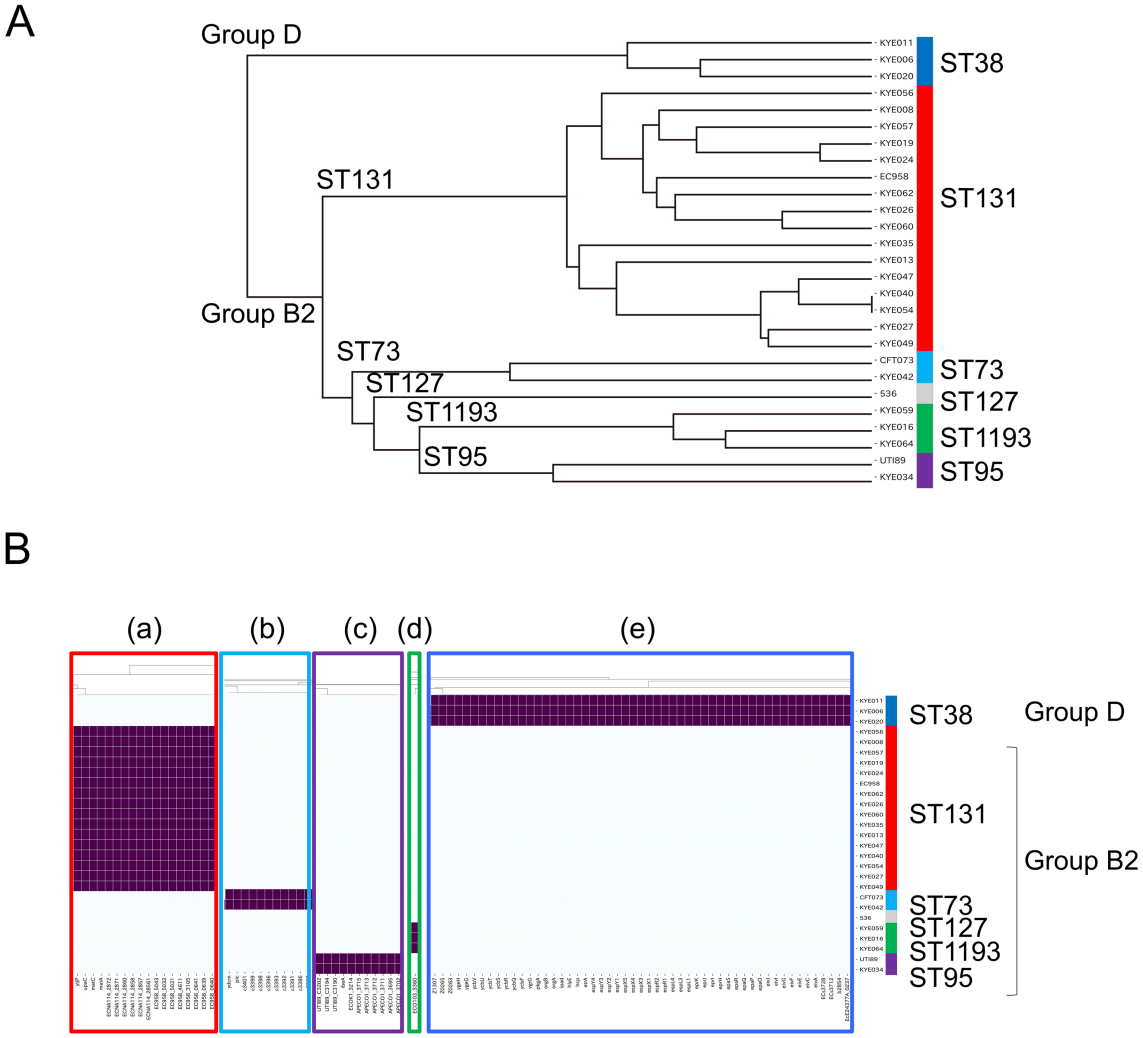
The ESBL-producing UPEC clinical isolates and representative UPEC strains clustered according to virulence-associated gene carriage detected by genome analysis. The presence of the gene is indicated with purple. Dendrograms of bacterial strains were deposited to distinguish the results **(A)**. The genes specifically detected in the ST131 (a), ST73 (b), ST95 (c), ST1193 (d), and ST38 (e) strains were shown **(B)**. Phylogroups B2 and D are indicated with Group B2 and D.

Cluster analysis of clinical isolates, including UPEC reference strains, was performed based on the presence of virulence genes, with each clade showing genotype-dependent clustering (Figure 1A). In terms of MLST-specific genes, the ST38 clinical isolates uniquely harbored genes associated with the *epa* (members of secondary T3SS-*epa*), *esp* (EPEC-T3SS secreted protein, T3SS-*esp*), *ycb*, and the *hlyE* (Figure 1B; Supplementary Table S4) genes. Notable, most of the ST131-, ST95-, ST73, and ST1193 specific genes were hypothetical (Figure 1B; Supplementary Table S4).

### 3.5 Homologs of virulence-associated genes and gene loci and their functional implications

The hypothetical genes identified within MLST-specific genes were analyzed using a BLAST search with amino acid sequences and gene order from the Prokka data. Based on the results from both ABRicate and Prokka, gene homologs or genes with multiple designations were further analyzed and annotated (Supplementary Table S5). Genes within the same operon or functional gene loci were subsequently grouped together (Supplementary Table S6).

Accordingly, T6SS gene members were found upstream and downstream of c3400, present in ST131 and other phylogroup B2 strains (Supplementary Table S7). T6SS is classified into three distinct groups: T6SS-1 to -3, based on their genetic structures. Nucleotide sequence analysis revealed that c3400 encoded *tssF* and *tssG* of T6SS-1. Other T6SS-1 gene homologs were consistently found around c3400 genes in other phylogroup B2 strains. Furthermore, the utilization of these homologs appeared to be MLST-dependent (Supplementary Table S7). The presence of T6SS-1 homologs was evalulated using the genomes of seven ST38 strains and 161 ST69 strains isolated from UTIs in EnteroBase (Supplementary Table S1). In contrast to the phylogroup B2 strains, c3400 and associated T6SS-1 homologs were absent in the genomes of ST38 and ST69 strains in EnteroBase (data not shown).

**Figure 2:**
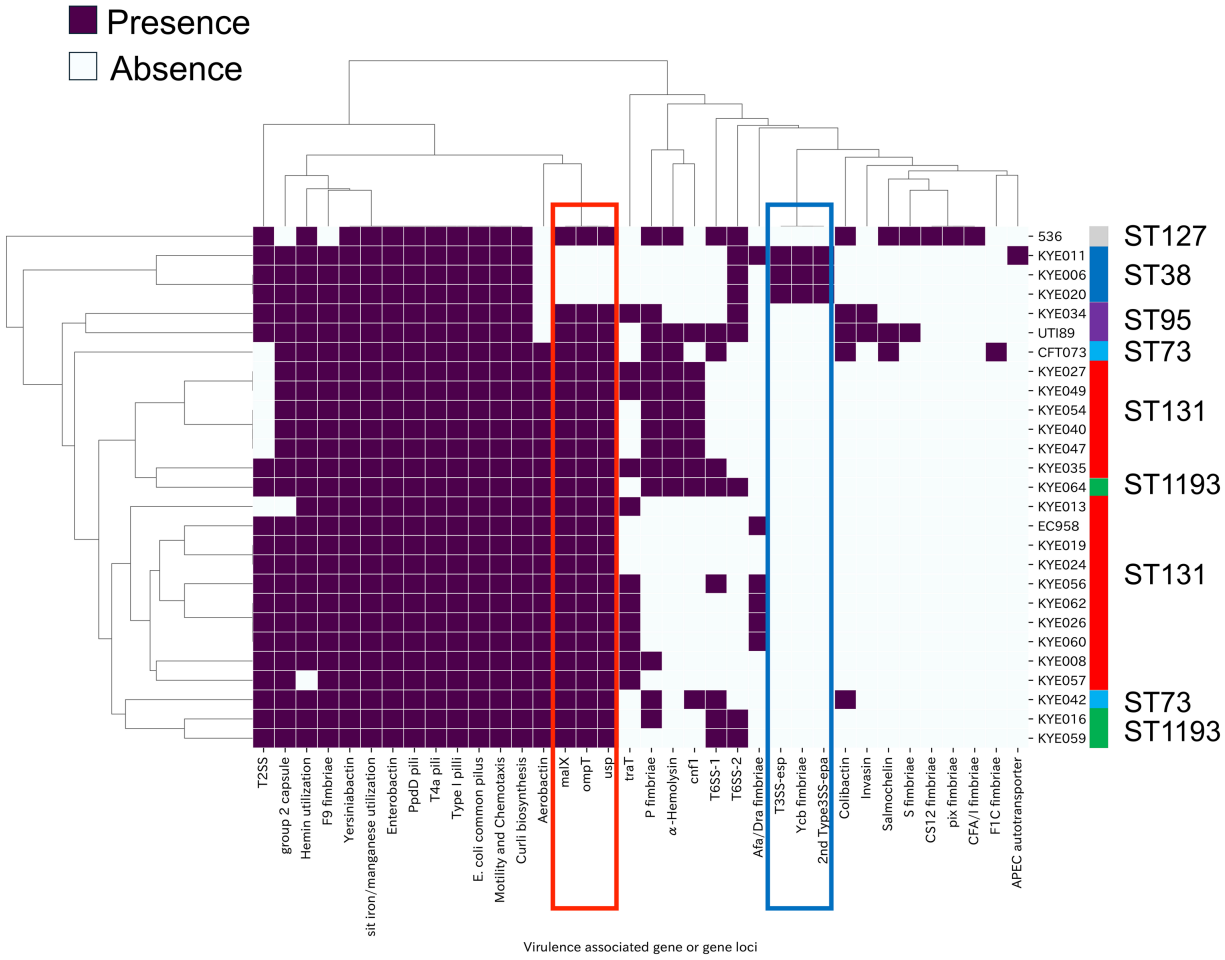
Hierarchical clustering of the bacteria based on the virulence-associated gene loci and genes profiles. Purple cells indicated the gene loci containing the predicted functional gene sets (Supplementary Tables S6, S8, S9). The genes that were specifically not detected in ST38 strains were indicated with a red box. The gene loci that were specifically detected in ST38 strains were indicated with a blue box.

Based on the gene loci (Supplementary Tables S6, S8, S9), clustering analysis was performed, including the four well-known virulence genes (*cnf1*, *ompT*, *traT*, and *usp*; Supplementary Table S9). The T6SS apparatus requires the construction of at least 13 genes for its assembly (Journet and Cascales, 2016; Navarro-Garcia et al., 2019). As previously reported, *tssM* was predictably absent in ST131-O25 strains (Cummins et al., 2023) and KYE034 (ST95) (Figure 2; Supplementary Tables S7–S9).

The *aec* genes were predicted to be members of the T6SS-2 family (Supplementary Table S7). With the exception of ST131, more than two of the T6SS-2 gene homologs were found in all strains, including ST38 (Supplementary Table S7). However, most T6SS-2 genes were lacking in KYE042 and also in CFT073 as shown in the previous data (Journet and Cascales, 2016). Therefore, functional T6SS-2 loci were absent in both ST131 and ST73 strains (Figure 2; Supplementary Tables S7–S9). Similar to T6SS-1 genes, the utilization of T6SS-2 gene homologs appeared to be MSLT dependent (Supplementary Table S7).

P fimbria, associated with pyelonephritis and UTIs (Ambite et al., 2019; Kuehn et al., 1992; Lane and Mobley, 2007), are encoded by 11 *pap* (pyelonephritis-associated pili) genes (Supplementary Table S6). Whole-genome analysis revealed that none of the *pap* genes were present in ST38 strains. In several phylogroup B2 strains, the *pap* operon was disrupted by an *IS* insertion downstream of *papI*, and these strains were negative for *papAH* and *papG* genes by PCR (Supplementary Tables S2, S8). Of the 38 ST131 isolates, 10 were positive for *papAH* and *papG*, suggesting they could likely express functional P fimbriae. This proportion was significantly lower compared to the non-ST131-phylogroup B2 isolates, where 4 out of 5 were positive for these genes (10 of 38; OR, 0.089; 95% CI, 0.0071–0.7031; *p* = 0.032).

Functional T3SS-*esp.,* the second T3SS-*epa*, Ycb fimbriae genes, and *hlyE* were found only in the ST38 strains (Figure 2; Table 8; Supplementary Tables S8, S9). ST69, belonging to phylogroup D, is frequently detected in UPEC strains. The presence of these genes was evaluated using the genomes of ST38 strains and ST69 strains (Supplementary Table S1). Accordingly, *ycb* genes were detected in all ST38 strains but were absent in the ST69 strains examined. In contrast, the second T3SS-*epa*, T3SS-*esp* genes, and *hlyE* were detected in all ST38 strains and in more than 90% of ST69 strains (data not shown).

**Table 8:** Presence and absence of virulence-associated gene or gene loci.

Classification	Gene loci	
Gene loci commonly detected in the ESBL-producing UPEC clinical isolates	Fimbriae	Type I pili Curli biosynthesis F9 fimbriae PpdD pili T4a pili *E coli* common pilus
	Iron utilization	Enterobactin Yersiniabactin Sit iron/manganese utilization (Hemin utilization)
	Motility	Motility and chemotaxis
	Immune evasion	(Group 2 capsule)
Phylogroup D-ST38 specific gene or gene loci	Fimbriae	Ycb fimbriae
	Secretion system	2nd T3SS-*epa* T3SS-*esp*
Gene or gene loci that were not specifically detected in phylogroup D-ST38	Fimbriae	P fimbriae
	Serum resistance	*ompT*
	UPEC PI marker	*malX*

The virulence-associated genes were grouped and indicated as gene loci as per to Supplementary Table S6. The gene loci found in all clinical isolates except for one strain were parenthesized.

Gene loci associated with attachment and colonization (type I pili, curli biosynthesis, F9 fimbriae, PpdD pili, T4a pili, *E. coli* common pilus), iron acquisition systems (enterobactin, yersiniabactin, and Sit iron/manganese utilization system), as well as motility, chemotaxis, and Group 2 capsules, were present in all strains except KYE013 (Figure 2; Table 8; Supplementary Table S9). Consequently, fewer secretion systems were detected in ST131 strains compared to non-ST131 strains (Table 9).

**Table 9:** Number of virulence genes gene loci detected in the clinical isolates examined.

	Phylogroup B2				Phylogroup D	
	ST131		Non-ST131		ST38	
Virulence genes and gene loci	Average	(Min-Max)	Average	(Min-Max)	Average	(Min-Max)
Adhesin (9)	6.7	(6-7)	6.8	(6-7)	7.3	(7-8)
Fe acquisition system (5)	4.9	(4-5)	4.8	(4-5)	4.0	(4-4)
Secretion system (5)	0.7	(0-2)	2.4	(1-3)	4.0	(4-4)
Others (6)	2.3	(1-3)	2.8	(2-4)	2.3	(2-3)

Data are expressed as average, and minitfnmum (Min), maximum (Max) number of gene loci consisting of functional gene members.

## 4 Discussion

The pathogenicity of ESBL-producing *E. coli* clinical isolates requires further elucidation; however, it remains insufficiently understood. In this study, we analyzed the genotypes and virulence-associated genes of ESBL-producing *E. coli,* focusing on strains that cause bloodstream infections, particularly those associated with UTIs.

Most clinical isolates were identified as ST131, with some belonging to ST73, ST95, and ST1193 in phylogroup B2. These findings are consistent with previous findings indicating that the most prevalent genotype among ESBL-producing UPEC strains consists of typical extraintestinal pathogenic *E. coli* (ExPEC) strains responsible for bloodstream infections (Clermont et al., 2013; Manges et al., 2019). ST73 and ST95 (Masui et al., 2022), known as “classic STs,” are highly prevalent in ExPEC, particularly when sample selection does not focus on AMR phenotypes (Gibreel et al., 2012; Yamaji et al., 2018; Fibke et al., 2019; Li et al., 2023). These STs are frequently associated with UTIs and bloodstream infections (Riley, 2014). ST1193, an emerging clone (Johnson et al., 2019), is linked to outbreaks and is imitating ST131 in terms of prevalence (Pitout et al., 2022; Tchesnokova et al., 2019). Although previous antimicrobial treatments may introduce bias, the high resistance rates observed in ST131 strains highlight the need for alternative therapeutic strategies (Table 4). ST38 is among the top 10 human pandemic lineages (Roy Chowdhury et al., 2023); however, it has been poorly investigated, with the exception of comprehensive phylogenomic analyses (Manges et al., 2019).

For the analysis of representative UPEC-specific genes by PCR, primers were designed for the *kpsMT*, *papAH*, and *papG* genes using homologous regions from available genomes (data not shown). The primers toned to be meticulously designed, considering the polymorphisms observed in bacterial surface protein genes. Accordingly, only three genes were detected in all the strains, while nine were not detected in the ST38 strain by PCR (Table 5; Supplementary Table S2). These results were somewhat contradictory, given the involvement of ST38 strains in invasive infections, prompting us to conduct whole-genome and *in-silico* analyses. The number of virulence-associated genes in ST38 strains was similar to that in the non-ST131 phylogroup B2 strains, and higher than that in the ST131 strains (Table 7; Supplementary Table S4). Further annotation and identification of gene loci to clarify the repertoire of virulence-associated genes highlighted the specificity of ST38 (Figure 2).

*papGII* has been identified as the only gene associated with invasive infections and severe UTIs (Lane and Mobley, 2007; Biggel et al., 2020). Despite the disruption of the locus in several B2 strains by *IS*, the P fimbriae gene was detected in all B2 strains. It is possible that some *pap* genes may have been lost during evolution, or that *pap*-deficient genes may have been horizontally transmitted in these strains. The ST38 strains were distinguished from the B2 clinical isolates based on the absence of P fimbriae genes and the presence of several EPEC genes (Figure 1B; Supplementary Tables S4, S8). These findings suggest that the ST38 strain, along with B2 strains that have incomplete *pap* genes, may rely on alternative factors for invasive infection.

The *ycb* operon has been found in some *E. coli* strains, but not all, and is present as a hidden system in the nonpathogenic *E. coli* strain K12 (Korea et al., 2010; Hsiao et al., 2016). The *ycb* operon plays a critical role in *E. coli* entry into HCT-8 cells, a human ileocecal epithelial cell line (Hsiao et al., 2016). Further gene function analyses will allow for further elucidation of Ycb expression and function in ST38 strains.

T6SS is a secretion system found in Gram-negative bacteria that is often associated with pathogenic strains and absent in nonpathogenic strains (Navarro-Garcia et al., 2019). Although the structure of the apparatus is conserved, the T6SS effector proteins vary. The T6SS in *E. coli* plays a role in the toxicity to bacterial and eukaryotic cells (Journet and Cascales, 2016).

Meanwhile, *in silico* analysis revealed the presence of the T6SS-1 gene locus near c3400 in all phylogroup B2 strains, but not in the ST38 strains (see Supplementary Tables S4, S7, S8). T6SS-1 is linked to enteroaggregative *E. coli* (EAEC) and avian pathogenic *E. coli* (Journet and Cascales, 2016), even though it is absent in phylogroup D strains, as observed in avian pathogenic *E. coli* (Tantoso et al., 2022). Overall, it is likely that several T6SS-1 genes are absent in the trains evaluated in the present study. Previous data have shown that the deletion of T6SS-1 genes in CFT073 do not affect colonization in the bladders and kidneys of CBA/J mice (Lloyd et al., 2009). Further analysis of T6SS expression is needed to understand the biological roles of these gene loci.

The functional T6SS-2 loci were found in all strains except for ST131 and ST73. *E. coli* T6SS-2 was found in EAEC and linked to phylogroups D to F, but not to B2 (Chen et al., 2021). Consistent with previous studies, T6SS-2 was also detected in ST95 strains (Tantoso et al., 2022). According to Prokka analysis, genes encoding Rhs-family proteins, predicted to be effector proteins, were found upstream of the *vgrG* gene in ST38 strains (data not shown) (Günther et al., 2022), suggesting the utilization of different effectors in these and other B2 strains. T6SS-2 affects colonization, survival, and invasion (Zhou et al., 2012). Consequently, the absence of the T6SS-2 locus in ST131 may have reduced its invasiveness.

Since the *E. coli*_VF database contains more virulence genes than the VFDB database, we used the *E. coli*_VF database for our analysis. The ST131 strains had fewer virulence-associated genes, particularly secretion system genes, compared to the non-ST131 strains (Table 9). ST131 strains are recognized as virulent UPEC clones, and are often isolated from invasive infections (Biggel et al., 2020). In contrast, ST131 isolates showed lower virulence in mouse models than other isolates (Johnson et al., 2012). In the present study, a lower frequency of sepsis cases was observed for ST131 strains, with the secretion system genes—associated with the pathogenicity of EPEC—being less abundant in the most prevalent genotype, ST131. This finding correlates with the number of sepsis cases, even with limited clinical isolates. Although the relationship between the number of virulence genes and disease severity remains unclear (Merino et al., 2020), the number of virulence-associated genes were affected by secretion system genes (Table 9).

The increase in ESBL-producing *E. coli* can be attributed to the growing difficulty in treating UTIs, which complicates patient management (Foxman and Brown, 2003; Flores-Mireles et al., 2015). In the present study, the repertoires of virulence genes were found to be associated with MLST, with notable differences between ST38 strains and other B2 group strains. ST38 NDM-5-producing *E. coli* isolates have caused an outbreak in the Czech Republic (Chudejova et al., 2024). Genomic characterization and pathogenicity of clinical ST38 isolates are essential to monitor future trends. Moreover, ST131 strains revealed a lower frequency of virulence-associated genes than the other strains, with fewer secretion system genes. These results corroborate previous findings and suggest that the pathogenicity may vary among ESBL-producing UPEC strains causing invasive infections. To our knowledge, this study is the first to suggest that ST131 strains may exhibit lower pathogenicity than non-ST131 strains, based on the analysis of the number of virulence-associated genes and clinical data. However, the limited number of strains—all collected from the suburbs of Tokyo—necessitates a cautious interpretation of these findings. Future studies employing larger and geographically diverse strains, together with comprehensive clinical data, can definitively assess this relationship. Overall, the diversity in virulence of ESBL-producing UPEC strains causing severe infections highlights the need for further investigations to develop more effective treatment strategies.

## Data Availability

The datasets presented in this study can be found in online repositories. The names of the repository/repositories and accession number(s) can be found in the article/Supplementary material.

## Footnotes

^1^ https://github.com/tseemann/shovill

^2^ https://github.com/tseemann/prokka

^3^ http://clermontyping.iame-research.center

^4^ https://github.com/tseemann/mlst

^5^https://cge.food.dtu.dk/services/SerotypeFinder/, https://cge.food.dtu.dk/services/CHTyper/

^6^ https://github.com/tseemann/abricate

^7^ https://github.com/phac-nml/Ecoli_vf

^8^ https://blast.ncbi.nlm.nih.gov/Blast.cgi?PROGRAM=blastp&PAGE_TYPE=BlastSearch&BLAST_SPEC=&LINK_LOC=blasttab&LAST_PAGE=blastn

^9^ https://seaborn.pydata.org
